# Characterization of the resistome in manure, soil and wastewater from dairy and beef production systems

**DOI:** 10.1038/srep24645

**Published:** 2016-04-20

**Authors:** Noelle R. Noyes, Xiang Yang, Lyndsey M. Linke, Roberta J. Magnuson, Shaun R. Cook, Rahat Zaheer, Hua Yang, Dale R. Woerner, Ifigenia Geornaras, Jessica A. McArt, Sheryl P. Gow, Jaime Ruiz, Kenneth L. Jones, Christina A. Boucher, Tim A. McAllister, Keith E. Belk, Paul S. Morley

**Affiliations:** 1 Department of Clinical Sciences, College of Veterinary Medicine and Biomedical Sciences, Colorado State University, Fort Collins, CO, USA; 2 Department of Animal Sciences, College of Agricultural Sciences, Colorado State University, Fort Collins, CO, USA; 3 Agriculture and Agri-Food Canada Research Centre, Lethbridge, AB, Canada; 4 Department of Population Medicine & Diagnostic Sciences, Cornell University, Ithaca, NY, USA; 5 Centre for Food-borne, Environmental Zoonotic Infectious Diseases, Public Health Agency of Canada, University of Saskatoon, Saskatchewan, Canada; 6 Department of Computer Sciences, College of Natural Sciences, Colorado State University, Fort Collins, CO, USA; 7 Department of Biochemistry and Molecular Genetics, School of Medicine, University of Colorado, Denver, CO, USA

## Abstract

It has been proposed that livestock production effluents such as wastewater, airborne dust and manure increase the density of antimicrobial resistant bacteria and genes in the environment. The public health risk posed by this proposed outcome has been difficult to quantify using traditional microbiological approaches. We utilized shotgun metagenomics to provide a first description of the resistome of North American dairy and beef production effluents, and identify factors that significantly impact this resistome. We identified 34 mechanisms of antimicrobial drug resistance within 34 soil, manure and wastewater samples from feedlot, ranch and dairy operations. The majority of resistance-associated sequences found in all samples belonged to tetracycline resistance mechanisms. We found that the ranch samples contained significantly fewer resistance mechanisms than dairy and feedlot samples, and that the resistome of dairy operations differed significantly from that of feedlots. The resistome in soil, manure and wastewater differed, suggesting that management of these effluents should be tailored appropriately. By providing a baseline of the cattle production waste resistome, this study represents a solid foundation for future efforts to characterize and quantify the public health risk posed by livestock effluents.

Livestock production effluent has been implicated in the transmission of antimicrobial resistant bacteria into aquatic, terrestrial and atmospheric ecosystems^1^,^2^,^3^. Regulation of livestock manure and wastewater is currently designed to limit levels of organic nutrients and coliform bacteria within ground and surface waters, and manured soils. Such regulations may not adequately control transfer of antimicrobial resistance genes (ARGs) from livestock facilities into the surrounding environment, particularly when antimicrobial drug residues are present in effluents^4^,^5^,^6^. However, significant increases in soil ARG levels have been observed after application of antimicrobial-free soil^7^,^8^, or when manure application rates are high or manure is improperly stored^9^. Furthermore, non-manured or “pristine” soil contains a diverse repertoire of ARGs, making it difficult to distinguish between natural and anthropogenically impacted ARG content^10^.

In North American cattle production systems, antimicrobials are used to treat, prevent and control disease, as well as to improve production efficiency. Antimicrobial use in extensive management settings (i.e., cow-calf production settings on pasture) is low, with 1.9% of US and 2.7% of Canadian beef cows reportedly treated with antimicrobials^11^,^12^. In contrast, >20% of US feedlot cattle receive antimicrobials for prevention of respiratory disease, >13% for treatment of active respiratory disease, and >70% for the prevention of liver abscesses^13^. Ionophores are the most common antimicrobial administered to cattle in North American feedlots, but they are not classified as medically important^14^,^15^. Macrolides, which are classified as critically important, are the second most commonly administered antimicrobial^13^,^15^. Most North American dairy operations (>94%) administer antimicrobials to prevent mastitis during the dry period, with >97% of this use attributed to beta-lactam antimicrobials, including first- and third-generation cephalosporins^16^. Pre-weaned dairy calves are most commonly treated for respiratory disease and diarrhea, with phenicols, cephalosporins, macrolides, sulfonamides, tetracyclines and aminoglycosides all used in approximately the same proportions^16^.

Given these use practices and evidence that antimicrobial drug residues in livestock effluent can contribute to elevated antimicrobial resistance (AMR) levels, it is important to improve our understanding of how current management systems may impact AMR transmission to the public. Culture-independent methods can contribute to this understanding, as the genetic dynamics of AMR are complex and pan-microbial. For instance, ARGs often move together in a networked fashion^17^,^18^,^19^, and can be exchanged between distantly-related bacteria^20^,^21^,^22^,^23^. Transfer rates among bacteria increase under conditions that induce stress, including antibiotic exposure^24^,^25^. Pan-microbial approaches enable access to this microbial resistance ecology and provide information on how livestock production practices influence the density and composition of ARGs (i.e., the resistome^26^). With this insight, practical mitigation strategies can be proposed to minimize the flow of ARGs into aquatic, terrestrial and atmospheric ecosystems.

However, very little is currently known about the cattle production resistome. The few published studies are descriptive in nature, utilizing samples taken from a small number of non-commercial animals^27^,^28^. A lack of basic study design information is a major impediment to execution of larger, inferential studies. For instance, the sample size needed to detect resistome differences between conventional and organic operations is currently not estimable because we do not have a baseline of normal resistome variability among different cattle. In addition, many livestock production factors may confound the relationship between antimicrobial use and the resistome, e.g., stocking density or ration formulation. However, to-date these potential factors have not been identified, making it difficult to control for confounders in study design.

Therefore, the goal of this study was to provide a description of the dairy and beef production resistome in raw feces, soil and wastewater, and to identify factors that commonly influence this resistome. A conventional and an organic dairy were chosen to investigate the impact of antimicrobial use practices on the resistome; a US and a Canadian feedlot were chosen to explore the impact of diet (US feedlots typically feed corn-based diets, while Canadian feedlots utilize barley); and a cow-calf ranch was selected to compare resistome differences between intensive (i.e., feedlot) and extensive (i.e., pastured) production systems.

## Results and Discussion

Sequencing produced over 1.05 billion sequencing reads across all samples, at an average of 30.8 million reads per sample (range 14.9–48.9 M, Supplementary Table S1). Trimming resulted in removal of 20.9% of reads (range 15–29%) and filtering of *Bos taurus* removed a further 0.38% (range 0.003–5.4%) of reads (Supplementary Table S1).

Across all samples, 212 128 reads aligned to 34 resistance mechanisms that confer resistance to 15 classes of antimicrobial drugs (Supplementary Table S2). The majority of reads aligned to genes that confer tetracycline resistance (70.0%), and within this class, 98% of reads aligned to ribosomal protection proteins with the largest representation in TetQ and TetW (45.6% and 23.2% of all reads aligning to tetracycline ribosomal protection proteins, respectively). Of the 30 samples that contained reads that aligned to ARGs within the resistance database, 27 contained TetQ and 22 contained TetW. Interestingly, these resistance groups were also the most prevalent in fecal samples collected from >1 000 humans as part of two metagenomic studies^29^,^30^, suggesting that they may be common in both agricultural and human populations. Studies across diverse agricultural ecosystems also document the ubiquity of tetracycline resistance genes^31^,^32^.

Reads aligning to the macrolide-lincosamide-streptogramin (MLS) class of antimicrobials comprised 10.9% of all resistance-aligned reads. Among MLS-aligned reads, 61.1% aligned to macrolide efflux pumps, 22.1% to Erm 23s rRNA methyltransferases, and 16.7% aligned to lincosamide nucleotidyltransferases. The mefA efflux pump was the most frequently identified mechanism within the macrolide class.

### Few resistance mechanisms were identified in samples collected from the cow-calf ranch

None of the sequencing reads obtained from the soil and wastewater samples collected from the cow-calf ranch aligned to the resistance database (n = 4). This finding is unexpected given previous reports of a diverse and abundant resistome in soils and water not impacted by human activity^33^,^34^,^35^. However, these studies utilized functional metagenomics, PCR and/or very deep sequencing to detect ARGs, which may be better able to detect very low-abundance ARGs compared to shotgun metagenomics. In addition, the use of functional metagenomics offers the potential for novel resistance gene discovery^36^. While deeper sequencing may have revealed the presence of resistance gene sequences in the pasture soil and water samples, sequencing depth (as well as DNA quantity and quality) was not significantly different when comparing feedlot, dairy and ranch samples, suggesting that extensive rearing methods may result in lower resistance gene levels compared to more intensive rearing methods such as feedlots. Alternatively, soil and wastewater ARGs on ranches may not be as geographically concentrated and therefore more samples may be needed to gain a representative portrait of the ranch environmental resistome. However, soil samples were collected from areas where cattle commonly congregated, and water was collected from a dugout, and therefore these samples represent the resistome of locations where ARGs were likely to be most concentrated. In addition, the two fecal samples collected from the pasture contained 2 resistance mechanisms in total, as compared to 4, 11, 7 and 21 in the conventional dairy, organic dairy, US and Canadian feedlot samples, respectively. This suggests that extensive rearing may promote fewer mechanisms of resistance overall. Previous studies have documented a significantly lower level of integrons (which commonly carry ARGs) in grass-fed compared to lot-fed cattle, although the content of the integrons did not differ substantially between the two groups^37^. Culture-based studies also have documented lower levels of resistance in generic *Escherichia coli* isolated from beef cows on pasture compared to feedlot cattle^38^,^39^,^40^,^41^. However, the feedlot and pastured cattle populations differ in many other aspects, including genetics, sex and age of the cattle, and therefore more controlled studies are needed to determine whether differences in AMR are driven by antimicrobial use. Such studies should also take into account the “dilution effect” of cattle on pasture, and the likelihood that environmental samples from such settings are comprised largely of soil or water that has not been impacted by cattle activity.

### Resistome composition and diversity differ between calves and adult cattle

**Figure 1:**
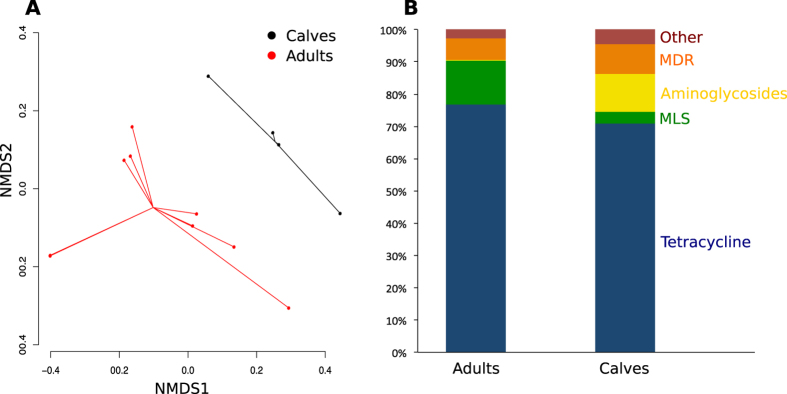
A comparison of fecal samples collected from calves and adult cattle. (**A**) NMDS ordination of fecal sample resistomes from calf vs. adult cattle. (**B**) Proportion of all aligned reads that aligned to ARGs within different resistance classes, in adult cattle versus calf feces. MDR = Multi-drug resistant mechanisms; MLS = Macrolide-lincosamide-streptogramin.

**Figure 2:**
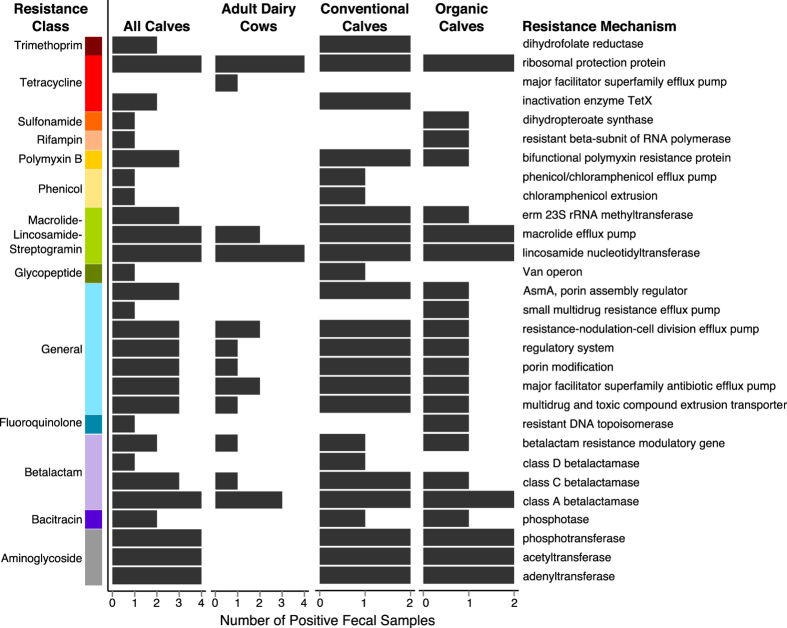
The calf fecal resistome is significantly more diverse and rich than the adult fecal resistome. Number of fecal samples containing alignments within each mechanism and class of resistance, separated by calves vs. adults, and conventionally raised vs. organically raised calves.

The resistome of fecal samples collected from preweaned dairy calves was different from that of mature cattle of all types (Stress = 0.11 and 0.03, ANOSIM R = 0.28 and 0.41, ANOSIM *P* = 0.05 and 0.02 at the mechanism and class levels, respectively, Fig. 1). Shannon’s diversity and richness at the mechanism and class levels were both lower in adult cattle compared to calf feces (Kruskal-Wallis *P* = 0.02 and 0.05 for richness and *P* = 0.09 and 0.09 for Shannon’s diversity, respectively). A descriptive comparison of dairy calf and adult dairy cow feces reflects this same pattern (Fig. 2), suggesting that production system (i.e., beef versus dairy) was not confounding this comparison and that the resistome of adult cattle feces is less diverse than that of calves. In addition, sequencing depth and DNA quantity and quality were not significantly different between samples collected from calves and samples collected from adult cattle, providing further evidence that resistome differences between these samples were not artifactual. Previous studies in calves have documented significant changes in fecal microbial diversity and composition as calves moved from milk-fed to weaning^42^,^43^ and these changes could be driving resistome composition.

Reads aligning to trimethoprim and aminoglycoside ARGs were identified in calf feces, but not in mature cattle feces (Fig. 2). This finding could reflect antimicrobial use practices in the conventional dairy, which administered trimethoprim-sulfadiazine and sulfamethazine to calves for treatment of scours, but did not administer these classes of antimicrobials to adult dairy cattle. Culture-based studies in generic *E. coli* report higher prevalence of resistance to trimethoprim-sulfamethoxazole and aminoglycosides (i.e., streptomycin, kanamycin and gentamycin) in beef calves compared to their corresponding dams^38^. However, aminoglycosides were not used in either dairy operation in this study, and therefore antimicrobial use practices do not directly explain these findings. A similar pattern regarding aminoglycoside resistance and use has been previously described in swine production, suggesting that aminoglycoside resistance may be co-selected by use of other antimicrobials^4^. We did not identify any tetracycline major facilitator superfamily (MFS) alignments in calf feces, but did in adult cattle feces, and reads aligning to macrolide efflux pumps and lincosamide nucleotidyltransferases were significantly more abundant in adult compared to calf feces. Lincosamides were used to treat mastitis on the conventional dairy farm, which could account for the increased lincosamide resistance in relation to preweaned calves. Alternatively, differences in resistome composition between calves and adult cattle could be driven by taxonomic differences in their respective fecal microbiomes. Studies in swine have shown significant shifts in the fecal microbiome as a function of age and diet, suggesting that the same patterns could be driving the differences observed in this study^44^,^45^. However, the degree to which microbiome drives resistome composition can vary dramatically^20^,^46^, and further research is needed to tease apart these potential confounders.

Comparison of the resistomes of conventionally- and organically-reared calves may shed light on the role of antimicrobial use practices in driving resistome differences. In this study population, organically raised calves did not receive any antimicrobial drugs, while the conventional dairy operation utilized beta-lactams (including cephalosporins), florfenicols, tetracyclines, macrolides, trimethoprim-sulfadiazine, sulfamethazine and macrolides. The 2 fecal samples collected from conventionally raised dairy calves contained slightly more unique resistance mechanisms (24/29) than those from organically raised dairy calves (22/29, Fig. 2). Among the 24 resistance mechanisms identified in feces from conventionally raised calves, 18 were identified in both conventional samples, while 7 of the 22 identified in feces from organically raised calves were identified in both organic samples (Fig. 2). These findings suggest that the resistome of organically raised calves may contain fewer unique resistance mechanisms and at lower frequency than the resistome of conventionally raised calves. However, the fecal resistome of organically raised calves contained a diverse set of resistance mechanisms, despite the naiveté of these calves to antimicrobial drugs. Similar findings have been reported in 6-month old infants that were unexposed to antimicrobials^47^, and culture-based studies of generic *E. coli* isolated from preweaned calf feces support the difference in AMR in conventional and organic calf feces^48^,^49^,^50^,^51^. Due to the stark difference in calf feces versus mature cattle feces, calf samples were removed from further analyses to enable a direct comparison of adult cattle feces.

### Beef feedlot resistome differs from the dairy resistome

**Figure 3:**
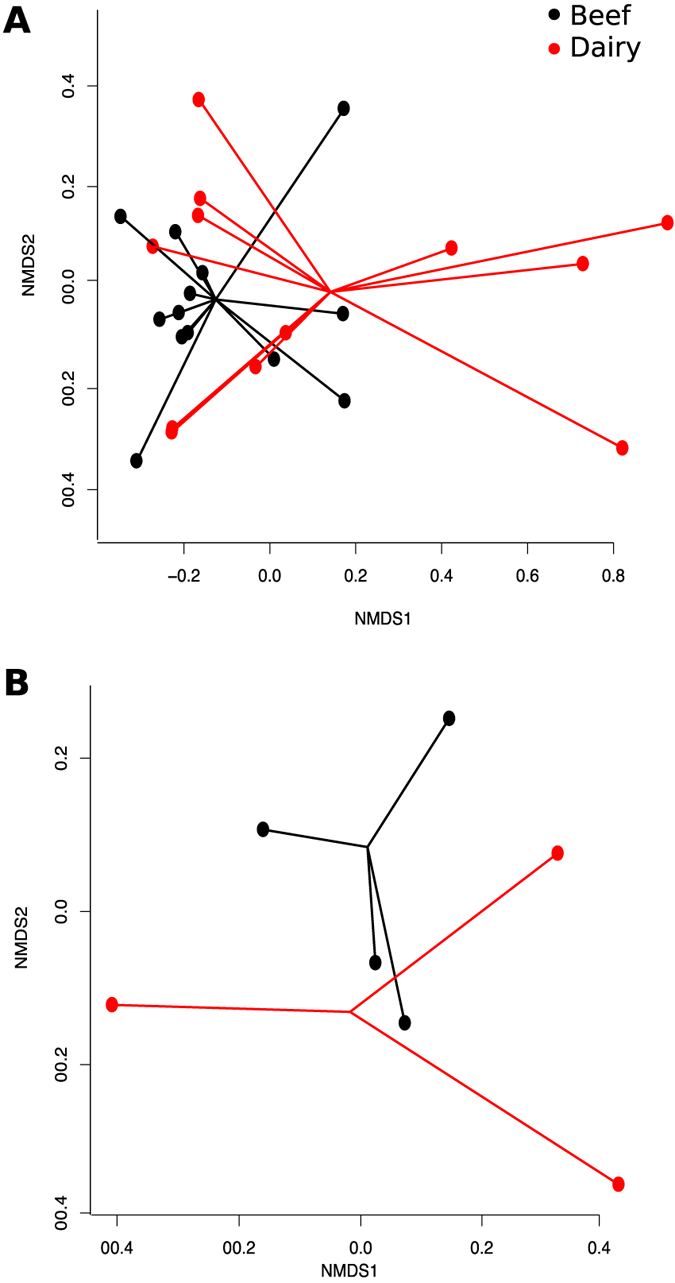
Beef and dairy systems have different resistomes. NMDS ordination at the mechanism level of (**A**) (adult) fecal, soil and wastewater samples and (**B**) only adult fecal samples were both significantly different based on system, e.g., beef vs. dairy (ANOSIM *P* < 0.05).

Resistome composition at the mechanism and class levels differed between beef (feedlot and pasture) and dairy operations, for all sample matrices (NMDS Stress = 0.11 and 0.06, ANOSIM R = 0.15 and 0.11, and ANOSIM *P* = 0.01 and 0.03, respectively, Fig. 3A). However, Shannon’s diversity and richness indices did not differ between beef and dairy cattle (data not shown). Sequencing depth and DNA quality and quantity did not differ significantly between beef and dairy samples. Within fecal samples, ordination at the mechanism level showed clear separation of beef and dairy resistomes (NMDS Stress = 0.006, ANOSIM R = 0.38 and ANOSIM *P* = 0.03, Fig. 3B); soil and wastewater resistomes of beef versus dairy could not be compared due to data sparseness. Of the 13 classes of resistance identified in all samples collected from adult cattle, 5 were more abundant in beef samples versus dairy, 2 were more abundant in dairy samples, 1 was nearly equally abundant in dairy and beef samples, and 5 did not pass filtering criteria due to low prevalence across samples. Even among classes that did pass filtering, several were present in very low abundance, and therefore estimates of log-fold differences in abundance may not be reliable. Consequently, the discussion is restricted to resistance classes present in at least 10 of the 30 soil, wastewater and adult fecal samples, i.e., tetracyclines, MLS, aminoglycosides, beta-lactams, and general-purpose mechanisms. The MLS resistance class was nearly equally abundant between dairy and beef samples. Alignments to tetracycline ARGs were more abundant in feedlots than in dairies, while aminoglycoside acetyltransferases and phosphotransferases were significantly more abundant in dairy samples. This pattern could reflect differential antimicrobial use practices in feedlots and dairies. Although the American Association of Bovine Practitioners strongly discourages dairy and beef veterinarians from administering aminoglycosides to cattle, nationwide surveys suggest that they are still being used with some frequency in dairies^16^, much less so in beef feedlots^13^. In addition, tetracyclines are used more frequently by US feedlots than US dairies, both in-feed and parenterally^13^,^16^. However, many other factors could account for these differences, including frequency of pen cleaning (which could influence the soil resistome), lagoon construction and management (which could impact the lagoon resistome), and feed composition (which could affect the fecal resistome). In addition, alignments to beta-lactam ARGs were more abundant in feedlot samples, even though these antimicrobial drugs are reported to be much more widely used in dairy production^13^,^52^. These findings highlight the complexity of dynamics between antimicrobial use and AMR, and suggest that use practices do not solely or directly influence AMR patterns in livestock production.

### Resistomes of feces, soil and wastewater are distinct

**Figure 4:**
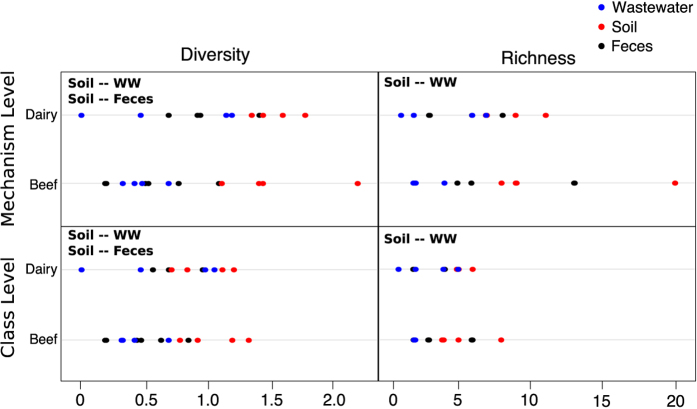
Soil samples are significantly more diverse and rich than wastewater. Dotplots showing Shannon’s diversity and richness at the mechanism and class levels, separated by system (beef vs. dairy) and colored by sample matrix, i.e., feces (black), soil (red) and wastewater (blue). Bolded text within each panel indicates which matrices differed based on Nemenyi post-hoc pairwise comparisons (WW = wastewater). Diversity and richness were not significantly different between beef and dairy at any level.

The resistome composition of feces, soil and wastewater were significantly different at the mechanism and class levels (NMDS Stress = 0.11 and 0.06, ANOSIM R = 0.34 and 0.30, and ANOSIM *P* = 0.001 and 0.001, respectively). Shannon’s diversity and richness were significantly higher in soil versus wastewater at the mechanism and class levels; however, soil samples received significantly more sequencing reads than water samples, and this could account for such differences^53^. Shannon’s diversity but not richness was significantly higher in soil versus feces at the mechanism and class levels (Fig. 4), and sequencing depth was comparable between the two matrices. The difference between soil and fecal diversity is especially interesting given that pen floors in dairies and feedlots are often a mixture of dried, compacted feces and underlying soil, rather than undisturbed soil matrix. In this context, the differentiation of “soil” (i.e., compacted feces) from fresh feces could stem from simple mixing of the two components, or from changes that occur within the soil-fecal matrix over time and under varying environmental exposures, as has been shown in *E. coli*^54^. Indeed, the fecal collection in this study focused on collection of recently voided fecal pats, in which exposure to aerobic conditions was of relatively short duration. Therefore, the environmental milieu to which the fecal and soil samples were exposed was likely very different.

**Figure 5:**
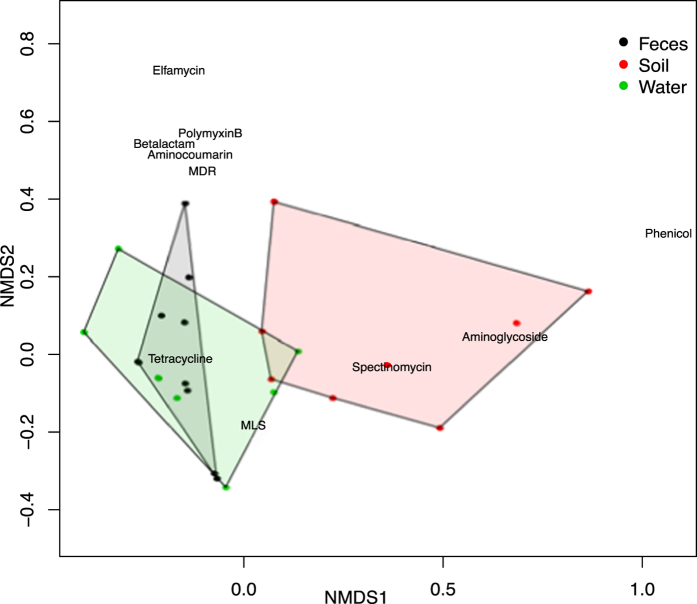
Specific resistance classes drive separation of soil from fecal and wastewater resistomes. NMDS ordination of fecal (black), soil (red) and wastewater (green) samples based on normalized counts of alignments aggregated at the resistance class level. Biplot coordinates of resistance classes are labeled with the class name, and show that aminoglycoside, phenicol and spectinomycin resistances differentiate the soil from the fecal and wastewater resistomes.

Ordination biplot results indicated that aminoglycoside, spectinomycin, phenicol and tetracycline resistance classes strongly influenced separation of soil samples from wastewater and feces (Fig. 5). Phenicol and spectinomycin resistance classes were not identified in adult cattle fecal samples, and reads aligning to aminoglycoside ARGs were less abundant in adult cattle fecal samples compared to soil samples (log-fold change = −4.6, adjusted *P* < 0.0001). Reads aligning to genes that confer tetracycline resistance–which were highly abundant in all samples–were more abundant in adult feces than in soil (log-fold difference = 1.50, adjusted *P* < 0.0001). This difference was driven overwhelmingly by the abundance of ribosomal protection proteins in feces, as major facilitator superfamily (MFS) efflux pumps were less abundant in feces compared to soil (log-fold difference = −2.1, adjusted *P* < 0.001). This pattern of tetracycline resistance mechanism differentiation between soil and feces has been observed in a comparison of human gut samples and agricultural soils^55^. Beta-lactam resistance alignments were also more abundant in feces and soil than in wastewater. Beta-lactams have been shown to degrade relatively quickly in the environment, and this could account for significantly lower levels in wastewater if antimicrobial drug residues influence the density of beta-lactam ARGs in this environment^56^.

The potentially confounding effect of sample matrix on resistome composition should guide future sampling efforts by providing a logical rubric on which to base future study designs. Fortuitously, wastewater, manure and soils are often managed separately in large livestock production systems, and therefore research efforts can focus within one matrix without forfeiting real-world applicability.

### Antimicrobial exposures on dairies did not significantly impact resistome composition

The resistomes of conventional and organic dairy samples (excluding calf fecal samples) were not different upon ordination, and there were no significant differences in Shannon’s diversity, richness or sequencing depth (*P* > 0.05). Abundance of resistance classes and mechanisms also did not differ, with the exception of macrolide resistance efflux pumps, which were more abundant in conventional dairy samples (log-fold difference = 9.4, adjusted *P* < 0.0001). The lack of differences may stem from insufficient study power, as descriptive analysis suggests major structural differences, particularly in the wastewater samples. For example, we identified only 2 resistance mechanisms in the organic dairy lagoon samples: lincosamide nucleotidyltransferases and tetracycline ribosomal protection proteins (specifically, TetQ and TetW). These samples clearly differed from the conventional dairy lagoon samples, which contained 9 resistance mechanisms. Previous studies have described significantly higher concentrations of TetO, TetW and Sul1 in lagoons at conventional dairies compared to organic dairies^57^, as well as higher levels of TetO, TetQ, TetW and TetM in feedlot lagoons draining pens exposed to moderate and high levels of antimicrobials as compared to lagoons draining antimicrobial-free pens^58^. Taken together, these findings suggest that antimicrobial use may substantially impact the resistome of stored wastewater, and therefore regulations regarding lagoon construction, wastewater use and management should be tailored to parameters of livestock operations, i.e., housing density or specific antimicrobial use protocols^59^. However, the influence of taxonomic composition on these resistome differences cannot be discounted, and such findings highlight the need for high-throughput methods that can definitively link microbiome composition with resistance genes. This is particularly important given recent studies reporting varying degrees of correlation between microbiome and resistome content, as well as varying rates of inferred horizontal gene transfer within microbial communities^20^,^46^. A shotgun metagenomic approach with short read sequence data can only provide a measure of microbiome-resistome correlation; such data do not enable definitive linking of resistance genes with the bacteria that harbor them. Long-read technology may represent a solution to this knowledge gap^60^, but relatively low throughput remains an obstacle for metagenomic applications.

Across soil, water and fecal samples, we identified 15 resistance mechanisms in non-calf conventional dairy samples and 18 in non-calf organic dairy samples. Reads aligning to class D beta-lactamases (bla-OXA), erm 23S rRNA methyltransferases, macrolide resistance efflux pumps, trimethoprim-resistant dihydrofolate reductases and the TetX inactivation enzyme were all identified in conventional but not organic samples. Conversely, organic samples contained several general-purpose resistance mechanisms not found in conventional samples (e.g., MATE, MFS efflux pumps and RND efflux pumps, porin modification genes, and regulators of resistance mechanisms). Resistance mechanisms with broad substrate specificity have been identified on organically-grown vegetables^61^, and our own research suggests that these types of mechanisms may be more abundant in cattle populations not exposed to antimicrobials. Under this theory, general purpose resistance mechanisms are favored in the absence of antimicrobial selective pressure as a means for bacteria to defend against numerous and varied environmental selective pressures, while resistance mechanisms with a more targeted purpose are favored in the presence of antimicrobial drugs. Further work is needed to test this hypothesis under more controlled settings.

### Resistome composition did not differ between US and Canadian feedlot samples

**Figure 6:**
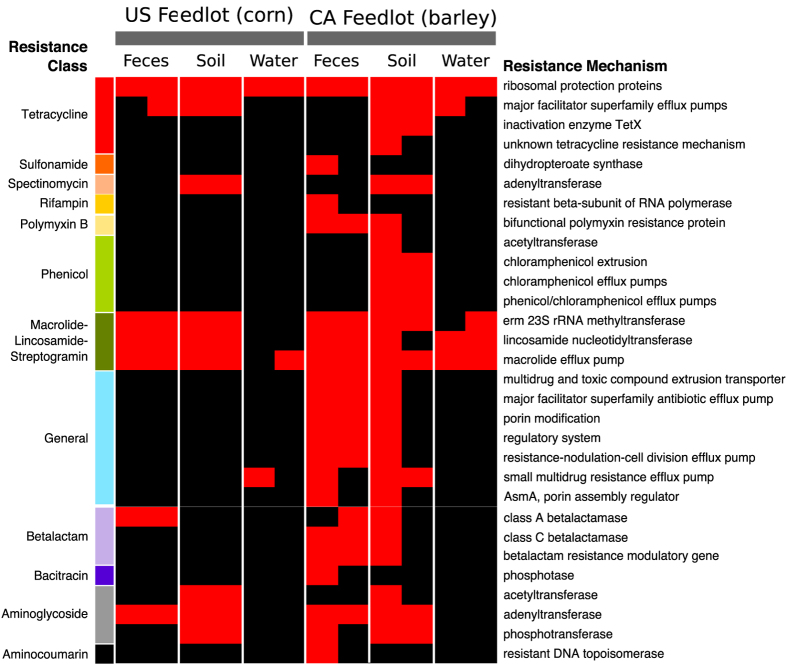
Binary heatmap of resistance mechanisms and classes identified in fecal, soil and wastewater samples collected from a US and a Canadian (CA) feedlot. Black = absent, red = present.

No significant resistome differences were observed between US and Canadian feedlot samples, which may indicate that diet did not have a major impact (NMDS Stress = 0.11 and 0.04, ANOSIM R = 0.02 and 0.05, and ANOSIM *P* = 0.31 and 0.26 for the mechanism and class levels, respectively). Diversity, richness and sequencing depth also did not differ significantly. However, descriptive results show differences, indicating that lack of statistical difference could stem from low study power (Fig. 6). Canadian samples contained reads that aligned to 12 classes and 30 mechanisms of resistance. In comparison, reads aligning to 6 classes and 11 mechanisms of resistance were identified in US feedlot samples. Previous studies have shown that diet significantly impacts the cattle fecal microbiome, which could impact the resistome^62^,^63^. While the US feedlot fed a corn-based and the Canadian feedlot a barley-based ration, antimicrobial use in these feedlots was largely similar (both feedlots administered in-feed tylosin) and this uniformity may have overpowered the influence of diet and microbial composition, leading to a lack of difference between samples.

### Utility of resistome analysis and implications for future studies

This pilot study compared the fecal, soil and wastewater resistomes of beef and dairy production. Using shotgun metagenomics, we revealed the diversity of the beef and dairy resistome through identification of 34 resistance mechanisms within 15 resistance classes. Wastewater, soil and pre-composted manure from intensive production operations contained varying types and levels of resistance mechanisms, and resistome composition was influenced by sample matrix, stage of animal development, and production system. These findings suggest that future research, regulations and decisions regarding livestock waste management should be tailored to effluent as well as to production system. Importantly, this study also highlights areas in which the shotgun metagenomics approach could be improved; specifically, longer reads could help to definitively identify the origin of resistance genes in metagenomic data and to link taxonomic differences with resistome differences. Finally, reference-based identification methods are *ipso facto* reliant on the accuracy and completeness of available databases. As with organisms in the microbiome, it is highly likely that many resistance mechanisms remain undiscovered, producing varying rates of false negatives and misclassification using available databases and bioinformatics tools^64^,^65^; our use of nucleotide homology for ARG identification may have also resulted in under-detection if the ARGs in the sample contained high rates of synonymous substitutions. Conversely, false positive identification can occur if sequences show high homology with known ARGs, but contain mutations that render them non-resistant. The necessary trade-off between sensitivity and specificity should be decided on a case-by-case basis, with deference to the goals of the study and full acknowledgement of limitations and potential misclassification. Regardless, as databases and classification methods continue to improve, so too should our ability to interpret results from metagenomic data. As this study demonstrates, resistome analysis can provide actionable guidance on complex agricultural AMR issues, and therefore improvement in associated methods and databases should be prioritized^66^,^67^. The results presented here provide a foundation for further research in this area, while also highlighting areas in which methodological improvements should be aggressively pursued.

## Methods

### Study population and sampling sites

Both dairies and the US feedlot were located in northeastern Colorado, while the Canadian feedlot and ranch were located in southern Alberta. The conventional dairy milked 990 cows on one location. Cows and calves were treated with a variety of antimicrobials for clinical illness and dry-off. The organic dairy milked ~16 500 mature cows daily and managed ~23 000 animals across 5 locations. No antimicrobials were used on the organic dairy. The US feedlot had a one-time holding capacity of ~90 000 head and fed cattle a steam-flaked corn-based diet. The Canadian feedlot had a one-time holding capacity of 17 000 cattle and fed a barley-based diet. Both the US and Canadian feedlots administered in-feed macrolides and ionophores to all cattle. The cow-calf ranch was located at the Onefour Agriculture Canada research station, which consisted of 17 000 hectare and carried ~600 cow-calf pairs. Less than one percent of the herd received antimicrobials, with treatment limited to a proportion of those individuals that exhibited clinical illness.

### Sample collection and processing

Sampling procedures were approved by the Colorado State University Institutional Animal Care and Use Committee, and were carried out in accordance with the Committee’s approved guidelines.

#### Feces

At each of the 5 operations described above, 2 composite fecal samples were collected from adult cattle. Additionally, 2 composite fecal samples from preweaned calves were collected at each of the 2 dairies, resulting in 14 total fecal samples for the study (10 adult and 4 calf). At the ranch, fecal samples were collected from an area where animals congregated at the time of sampling. Fecal samples at the feedlots and dairies were collected from 2 purposively selected pens. Feedlot pens identified as ready for slaughter were chosen such that samples represented cattle that had been >165 days-on-feed. At dairies, cows were grouped by level of milk production, and pens with highest production were chosen. To collect composite samples, personnel walked through pens or pasture area in a prescribed pattern, collecting ~1–2 grams (g) of freshly voided feces from 20 fecal pats. These 20 individual samples were placed in a sterile bag and thoroughly mixed by hand for 1 min, after which enough fecal material was removed to fill a sterile 50 milliliter (ml) Falcon tube. Dairy calves were housed in individual hutches, and therefore fecal samples were collected from the floor of 20 randomly selected individual hutches and mixed together as described above.

#### Soil

The same feedlot pens, dairy pens and pasture areas sampled for feces were also sampled for soil, using methods described for compositing of feces. Soil was not collected from dairy calf hutches, and therefore a total of 10 composite soil samples were collected (2 at each operation).

#### Wastewater

At each operation, 2 wastewater samples were collected. In the feedlots and dairies, wastewater was collected from the holding lagoon closest to the pens sampled during fecal collection. If lagoons were separated into “high-solids” and “low-solids”, a single sample was collected from each, and they were collected near the bank from opposite sides. Winter weather at the US feedlot prevented access to the entire lagoon, and therefore both samples were collected from the same side. At the cow-calf ranch, samples were taken from opposite sides of a dugout that cattle could fully access for drinking water.

### Sample processing and sequencing

Samples were placed on ice for transport to the laboratory. Fecal and soil samples were stored at −80 °Celsius (C) until processed. Water samples were stored at 4 **°**C for a maximum of 24 hours (h), and then centrifuged at 15 000 × g for 20 minutes (min). The resulting supernatant was decanted and each pellet was stored in a 15 ml Falcon tube at −80 **°**C.

After thawing, fecal and soil samples were pre-sedimented, allowing for processing of a greater sample volume (up to 10 g) while simultaneously removing PCR inhibitors known to be present in soil and feces. Briefly, 30 ml of buffered peptone water (BPW) was added to 10 g of soil or feces in a 50 ml conical tube and samples were shaken vigorously before allowing to sediment for 10 min. Supernatants, including limited soil/fecal debris, were transferred to a new 50 ml conical tube and centrifuged for 10 min at 4 300 × g. The BPW was removed and the resulting sample pellet was rinsed with 5 ml of molecular grade sterile PBS, and centrifuged again at 4 300 × g for 10 min. The supernatant was removed and the resulting pellet was resuspended in 15 ml of PowerBead solution before being processed with the Mo Bio PowerMax Soil DNA Isolation Kit (Mo Bio Laboratories, Solana Beach, CA, USA) according to manufacturer’s instructions. Water samples (250 milligrams [mg]) were processed using the Mo Bio PowerFecal DNA Isolation Kit according to manufacturer’s instructions. Extracted DNA from fecal, soil and wastewater samples was eluted in 400 microliters (μl), 200 μl and 50 μl of elution buffer, respectively. After extraction, DNA concentration was measured at 260 nanometers (nm) using a NanoDrop^TM^ spectrophotometer (Thermo Fisher Scientific, Waltham, Massachusetts, USA). Samples that were not sufficiently concentrated (defined as <20 nanograms/μl [ng/μl]) were subjected to ethanol precipitation to achieve a concentration of >50 ng/μl. Briefly, 1/10 volume of 3 molar (M) sodium acetate, pH 5.2, and 2 volumes of cold 100% molecular grade ethanol were added to the sample, which was then incubated at −20 °C for 1 h and centrifuged at 11 000 × g for 20 min at 4 °C. Supernatant was discarded and 150 μl of 70% cold ethanol was added; samples were centrifuged a final time at 11 000 × g for 10 min at 4 °C. Supernatant was again discarded and the DNA pellets allowed to air dry before resuspending in ¼ the original volume.

After DNA extraction and ethanol precipitation, samples were checked for purity using a NanoDrop^TM^ spectrophotometer (Thermo Fisher Scientific); all samples attained a 260:280 ratio of >1.4. Finally, ≥10 μl of DNA from each sample was delivered for library preparation (according to manufacturer’s protocol) and single-end sequencing on the Ion Proton^TM^ platform using the P1 chip (Thermo Fisher Scientific).

### Bioinformatics

Reads were trimmed and filtered for quality^68^: the leading 3 and trailing 3 nucleotides were removed, then nucleotides were removed from the 3′ end until the average Phred score across the first 4 nucleotides was at least 15. Sequences with <36 nucleotides were discarded. Adaptors were removed using a maximum of 2 mismatches in the initial seed, and adapter clipping occurred if a match score of 30 was reached. Upon clipping, both reads of a pair were retained to supply more reads for downstream applications. Reads classified as host (i.e., bovine) were removed by alignment to the UMD 3.1 *Bos taurus* genome with BWA using default parameters^69^. Non-host reads were then aligned to a custom non-redundant database of 3 111 ARG sequences compiled from the actively curated ARG databases ARG-ANNOT^70^, Resfinder^71^ and CARD (all downloaded on August 12^th^, 2014)^72^ using BWA with default settings. These databases were chosen because they are specific to antimicrobial resistance genes, they contain nucleotide sequences (as opposed to amino acid sequences), and they are actively curated and frequently updated. Redundant sequences between ARG-ANNOT and Resfinder were identified using CD-HIT-EST-2D^73^ with local alignment (-G 0) and the following parameters: -c 1.0 -AS 0 -AL 0 -aL 1.0 -aS 1.0. A single representative sequence was selected from each resulting cluster (n = 1,427), and these sequences were appended to the list of unique gene sequences in ARG-ANNOT (n = 261) and Resfinder (n = 715). This process was then repeated for the CARD database using the combined ARG-ANNOT/Resfinder non-redundant database. Seven hundred and eight sequences were unique to CARD, resulting in a final non-redundant database containing 3,111 unique ARG sequences. ARGs with >80% gene fraction (i.e., >80% of the nucleotides in the ARG sequence were covered by at least one read) were considered to be positively identified in a sample. Reads that aligned to ARGs that confer resistance via substitutions, insertions and/or deletions within antibiotic drug target genes were manually verified to match the reference ARG with 100% amino acid homology across 100% of the middle 95% of the ARG sequence (the very ends of the sequence were not included as reads rarely align to these “overhang” regions); synonymous substitutions were permitted. This list comprised the gene groups gyrA and parC (fluoroquinolone resistance), parE (aminocoumarin resistance), rpoB (rifampin resistance), folP (sulfonamide resistance), dfr (trimethoprim resistance) and EF-Tu (elfamycin resistance). This post-processing step was necessary in order to prevent potential false positives due to the presence of “wild-type” genes (i.e., genes not containing the resistance-conferring mutation) within the metagenomic data. Such reads could align to the reference ARG sequence with near-perfect identity, thus resulting in false identification of ARGs at the >80% gene fraction cutoff. Manual verification was conducted by visually inspecting alignment results using Tablet^74^. Alignments that did not pass post-processing verification were not included in downstream analyses.

### Data Analysis

Samples without identified ARGs (n = 4) were removed from further analysis due to an inability to normalize and ordinate. Alignments to each positively identified ARG were summed within sample and normalized using a data-driven approach based on shifts in count distributions^75^,^76^. Because conventions for naming ARGs based on sequence homology vary between resistance classes, normalized counts were aggregated to the mechanism and class level to generate a less biased comparison among samples.

Primary comparisons included resistome differences between beef and dairy systems; conventional and organic dairy samples; calves and adult cattle; pasture and intensively managed operations; and between Canadian and US feedlots. In addition, we investigated differences in sample matrix (e.g., feces vs. soil vs. wastewater).

Non-metric multidimensional scaling (NMDS) using Euclidean distances between Hellinger-transformed normalized counts was used to ordinate samples based on resistome composition^77^. Significant dissimilarity of ordination between groups was assessed using the Analysis of Similarities statistic^78^.

To compare Shannon’s diversity and richness across groups, as well as to assess potential sequencing bias, a Kruskal-Wallis test statistic with Nemenyi post-hoc comparisons adjusted for rank ties was used. Separate comparisons were made between system (beef vs. dairy vs. pasture), sample matrix (feces vs. soil vs. water), dairy production type (organic vs. conventional) and feedlot diet type (corn vs. barley).

To assess differences in abundance of resistance alignments at the mechanism and class levels while accounting for potential uneven sequencing depth, zero-inflated Gaussian mixture models were used^75^. Prior to modeling, features (i.e., classes or mechanisms) present in fewer than 3 samples were removed from the dataset, as sparse features create model instability and do not provide reliable model estimates. When applicable, sample matrix and/or system (e.g., beef versus dairy) were added to models as potential confounders. Pairwise comparisons of log-fold change in abundance between groups were conducted using limma^79^, adjusting for multiple comparisons using the Benjamini-Hochberg procedure and a critical α of 0.05^80^.

## Additional Information

**Accession codes**: The sequence information in this paper has been submitted to the GenBank Sequence Read Archive database under BioProject accession number SRP067931. 

**How to cite this article**: Noyes, N. R. *et al.* Characterization of the resistome in manure, soil and wastewater from dairy and beef production systems. *Sci. Rep.* **6**, 24645; doi: 10.1038/srep24645 (2016).

## Supplementary Material

Supplementary Table S1

Supplementary Table S2
